# Effects of *Rhododendron mucronulatum* Branch Extract on Dexamethasone-Induced Muscle Loss and Muscle Atrophy in C57BL/6 Mice

**DOI:** 10.3390/ijms27219811

**Published:** 2026-11-01

**Authors:** Tae Hee Kim, Ye Eun Kwon, Hyeon Du Jang, Da Hyeon An, Eun Ji Kim, Jae In Jung, Sangil Min, Tae Hyung Kwon, Hee Kyu Kim, Jae Hun Jeong, Seung-Dai Kim, Sun Eun Choi

**Affiliations:** 1 Dr.Oregonin Inc., #802 Bodeum Hall, Kangwondaehakgil 1, Chuncheon 24341, Gangwon State, Republic of Korea; kth02120@naver.com (T.H.K.); kye0519@naver.com (Y.E.K.); 2 Department of AI Biological Parts Materials Engineering, Graduate School, Nambu University, Gwangju 62271, Republic of Korea; sdkim@nambu.ac.kr; 3 Department of Forest Biomaterials Engineering, Kangwon National University, Chuncheon 24341, Gangwon State, Republic of Korea; wkdgusen98@naver.com (H.D.J.); 202416560@kangwon.ac.kr (D.H.A.); 4 Research Institute, SRE Service Co., Ltd., Chuncheon 24232, Gangwon State, Republic of Korea; myej44@naver.com; 5 Institute of Medical Bio-Convergence, Hallym University, Chuncheon 24252, Gangwon State, Republic of Korea; jungahoo@hallym.ac.kr; 6 Division of Transplantation and Vascular Surgery, Department of Surgery, Seoul National University Hospital, Seoul 03080, Republic of Korea; surgeonmsi@gmail.com; 7 Department of Barista and Bakery, Gangwon State University, Gangneung 25425, Gangwon State, Republic of Korea; taehyung0218@gw.ac.kr; 8 Gangwon State Forest Science Institute, 24, Hwamokwon-gil, Chuncheon 24207, Gangwon State, Republic of Korea; dearkyu@korea.kr; 9 Division of Food Science & Biotechnology, Faculty of Agri-Food, College of Future Challenges, Mokpo National University, Damyang 57337, Jeollanam-do, Republic of Korea; jhjeong@mnu.ac.kr

**Keywords:** dexamethasone-induced muscle atrophy, *Rhododendron mucronulatum*, taxifolin-3-O-arabinopyranoside, Akt/mTOR/FoxO3α, young adult male C57BL/6 mice

## Abstract

We investigated the effects of *Rhododendron mucronulatum* branch extract (RMB) on dexamethasone (DEX)-induced muscle atrophy in young adult male C57BL/6 mice. Mice received oral RMB (100, 200, or 400 mg/kg) for 4 weeks and intraperitoneal DEX (5 mg/kg) during the final 2 weeks. RMB significantly improved exercise duration and capacity at 200 and 400 mg/kg (400 mg/kg vs. DEX control: time to exhaustion, 29.5 vs. 21.3 min), and grip strength showed an increasing trend. Muscle fiber cross-sectional area and gastrocnemius weight were preserved at all doses. RMB increased the phospho-Akt/Akt ratio at 200 mg/kg, the phospho-mTOR/mTOR ratio at 400 mg/kg, and the phospho-FoxO3α/FoxO3α ratio at all doses. *Atrogin-1* was suppressed at all doses and *MuRF1* at 400 mg/kg, whereas *MyoD* and *Myogenin* were increased at 400 mg/kg and *IGF-1* at 200 and 400 mg/kg. RMB also reduced intramuscular *TNF-α* and *IL-6*, restored antioxidant defense in muscle and serum, and increased Bcl-2. These findings suggest that RMB ameliorates DEX-induced muscle atrophy mainly by partially restoring Akt/mTOR signaling, promoting FoxO3α phosphorylation, and suppressing *Atrogin-1* and *MuRF1*, with additional anti-inflammatory, antioxidant, and anti-apoptotic effects.

## 1. Introduction

Skeletal muscle is not only the organ of locomotion but also communicates with other organs through secreted myokines [1,2,3,4], and this muscle–organ crosstalk has been linked to chronic conditions such as periodontal disease and Alzheimer’s disease [5,6,7,8]. Skeletal muscle is also a major regulator of whole-body metabolism, including glucose homeostasis, insulin sensitivity, and thermoregulation [9,10,11,12,13,14]. Sarcopenia, the progressive age-related loss of skeletal muscle mass and strength, impairs mobility, increases fracture risk, and reduces the capacity to perform activities of daily living [15,16,17,18,19,20], and it is increasingly regarded as a condition with consequences beyond the musculoskeletal system [4].

Muscle loss results from an imbalance between protein synthesis and degradation, together with chronic inflammation, hormonal changes, neuromuscular junction damage, and mitochondrial dysfunction [21]. In particular, excessive reactive oxygen species (ROS) and weakened antioxidant defenses promote oxidative damage and apoptosis in myofibers, as reflected by a decreased B-cell lymphoma 2 (Bcl-2)/Bcl-2-associated X protein (Bax) ratio [22]. In addition, anabolic signaling through insulin-like growth factor-1 (IGF-1), protein kinase B (Akt), and mammalian target of rapamycin (mTOR) is suppressed, whereas forkhead box O3α (FoxO3α) promotes the expression of muscle-specific E3 ubiquitin ligases Atrogin-1 and muscle RING finger-1 (MuRF1) [23,24,25].

Resistance exercise and protein supplementation are the first-line interventions for sarcopenia [26,27,28], and hormonal agents such as growth hormone, testosterone, and oxymetholone have also been investigated [29,30,31]. However, exercise and hormonal therapies are not always effective, excessive animal-protein intake has been associated with potential renal and hepatic effects in some contexts [32,33,34], and long-term use of oxymetholone has been associated with hepatotoxicity [35,36]. Given these limitations, plant-derived polyphenols have attracted interest as candidate agents for muscle atrophy; however, as with synthetic compounds, their efficacy and safety must be established empirically. Polyphenols have been reported to attenuate inflammation and oxidative stress in skeletal muscle, including the modulation of interleukin-6 (IL-6) and tumor necrosis factor-α (TNF-α), to regulate protein degradation pathways, and to support mitochondrial function, thereby contributing to the maintenance of muscle mass and function [37,38,39,40,41,42,43,44,45,46,47,48,49,50,51,52,53,54,55,56,57].

*Rhododendron mucronulatum*, a shrub native to Korea, contains diverse polyphenols and flavonoids in its roots, branches, leaves, and flowers [58,59] and has been used in traditional Korean medicine for respiratory complaints, arthritis, detoxification, and blood circulation [60,61]. Our chemotaxonomic studies of the genus *Rhododendron* identified taxifolin-3-O-arabinopyranoside (Tax-G), a glycoside of taxifolin (dihydroquercetin), as a compound particularly abundant in *R. mucronulatum* [62,63,64], and taxifolin-type flavonoids have well-documented antioxidant and anti-inflammatory activities [65]. We and others have reported anti-atopic, anti-inflammatory, and anti-hair-loss activities of *R. mucronulatum* extract, including the regulation of apoptosis in human cells [66,67,68].

We previously demonstrated the anti-atrophic effects of *Ulmus macrocarpa* and *Alnus japonica* extracts in vitro and in vivo [39,40,41,42], and showed that *R. mucronulatum* branch extract (RMB), Tax-G, and taxifolin protect C2C12 myotubes against dexamethasone (DEX)- and H_2_O_2_-induced injury by regulating Akt/mTOR/FoxO3α signaling and apoptosis-related proteins [43]. However, cell-based findings cannot establish whether orally administered RMB preserves muscle mass, strength, and endurance in a whole organism, nor whether its effects are dose-dependent. Therefore, the present study aimed to determine whether oral RMB (100–400 mg/kg) attenuates DEX-induced muscle atrophy in young adult male C57BL/6 mice. To this end, we assessed functional performance (treadmill endurance and grip strength); body composition; muscle mass and fiber size; and intramuscular signaling, gene expression, and systemic antioxidant markers selected in accordance with the functional food guidelines of the Korean Ministry of Food and Drug Safety (MFDS).

## 2. Results

### 2.1. Effects on Body Weight of Experimental Animals

**Figure 1:**
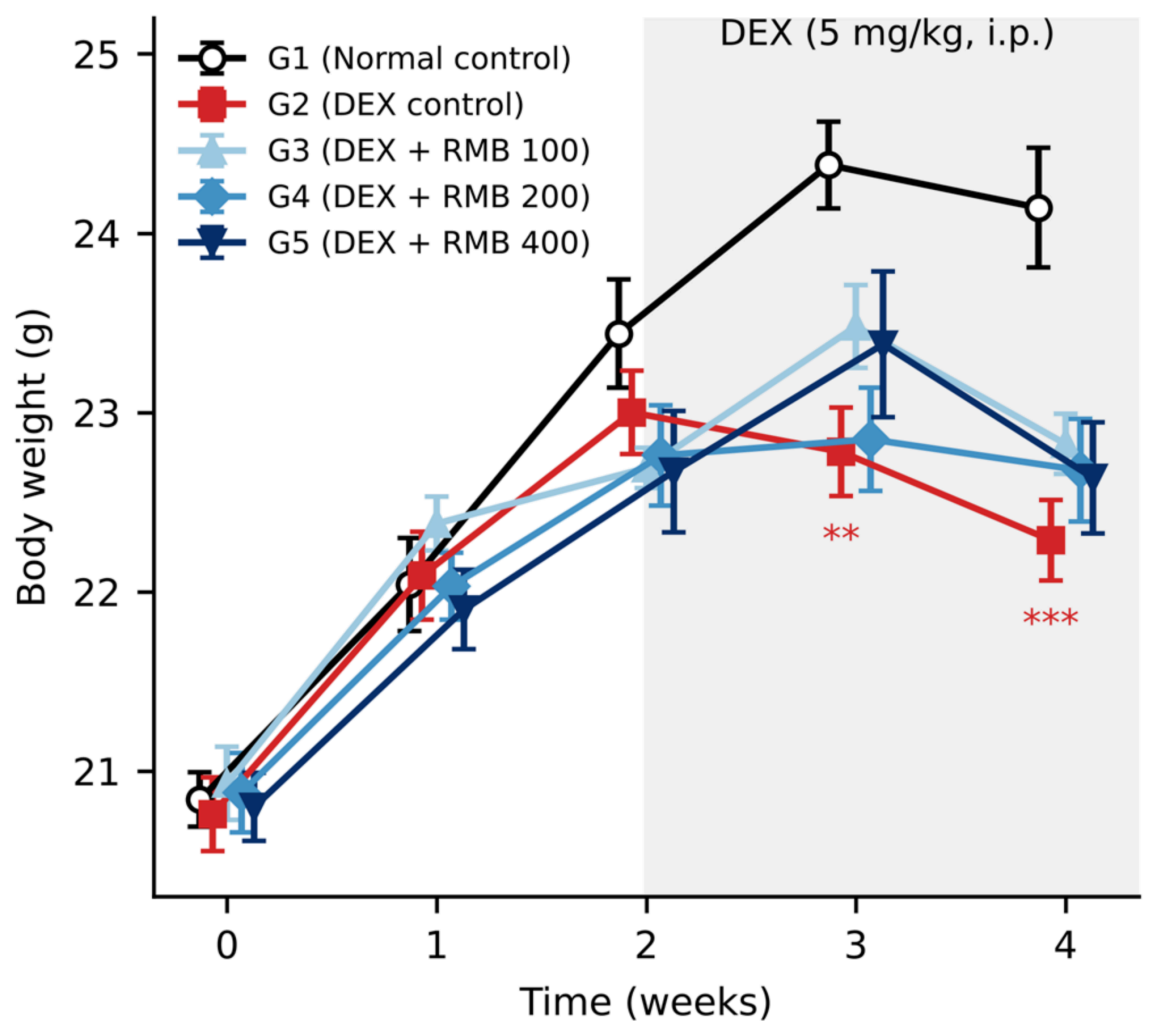
Body weight of experimental animals during the study. Values are expressed as mean ± standard error of the mean (SEM) (*n* = 10 per group). ** *p* < 0.01, *** *p* < 0.001 vs. G1 (one-way ANOVA followed by Bonferroni-corrected pairwise comparisons). G1, normal control; G2, DEX control; G3–G5, DEX + RMB 100, 200, and 400 mg/kg.

Body weight was measured weekly throughout the study (Figure 1). The normal control group (G1) gained weight steadily up to week 3, after which its body weight plateaued with a slight decrease in the final week, whereas body weight in the DEX control group (G2) was significantly lower than in G1 from week 3 onward, consistent with DEX-induced muscle wasting. No significant differences in body weight were observed between G2 and the RMB-treated groups (G3–G5) (Figure 1).

### 2.2. Effects on Exercise Time and Exercise Capacity

**Figure 2:**
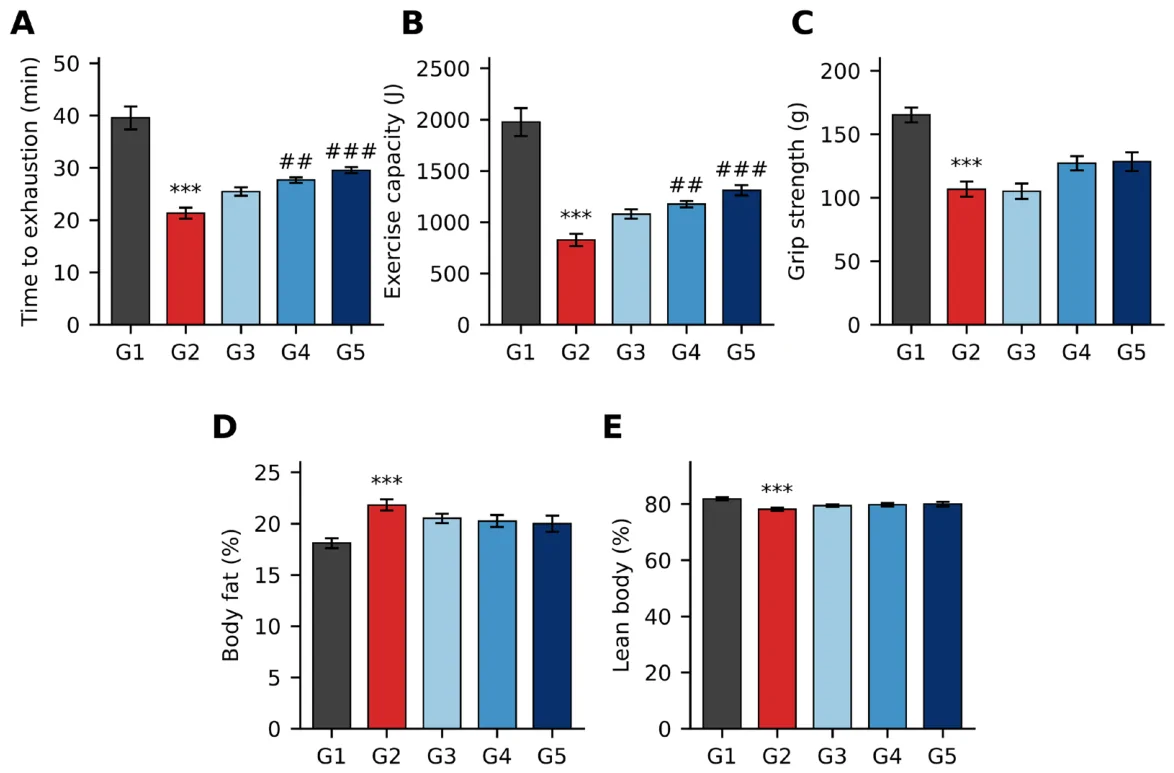
Effects of RMB on physical performance and body composition. (**A**) Time to exhaustion, (**B**) exercise capacity, (**C**) grip strength, (**D**) body fat percentage, and (**E**) lean body percentage. Values are expressed as mean ± SEM (*n* = 10 per group). *** *p* < 0.001 vs. G1; ## *p* < 0.01, ### *p* < 0.001 vs. G2 (one-way ANOVA followed by Bonferroni-corrected pairwise comparisons). G1, normal control; G2, DEX control; G3–G5, DEX + RMB 100, 200, and 400 mg/kg.

As shown in Figure 2A, time to exhaustion was significantly shorter in the DEX control group (G2; 21.3 ± 1.0 min) than in the normal control group (G1; 39.6 ± 2.2 min) and was significantly increased in G4 (27.7 ± 0.5 min) and G5 (29.5 ± 0.6 min) compared with G2, and also tended to increase in G3 (25.5 ± 0.8 min). Exercise capacity was likewise significantly reduced in G2 (826.4 ± 59.7 J) compared with G1 (1975.8 ± 137.3 J) and was significantly improved in G4 (1176.6 ± 31.2 J) and G5 (1310.8 ± 49.7 J), with an increasing trend in G3 (1078.8 ± 45.5 J) (Figure 2B).

### 2.3. Effects on Grip Strength

Grip strength was significantly lower in G2 (106.7 ± 5.9 g) than in G1 (165.2 ± 5.9 g). RMB treatment tended to increase grip strength in G4 (127.2 ± 5.5 g) and G5 (128.4 ± 7.3 g) compared with G2 (Figure 2C).

### 2.4. Effects on Fat Percentage and Lean Body Percentage

DEX significantly increased body fat percentage (G1, 18.1 ± 0.5%; G2, 21.8 ± 0.5%) and decreased lean body percentage (G1, 81.9 ± 0.5%; G2, 78.2 ± 0.5%). RMB tended to reverse these changes in a dose-dependent manner (Figure 2D,E).

### 2.5. Effects on Muscle Weight

**Figure 3:**
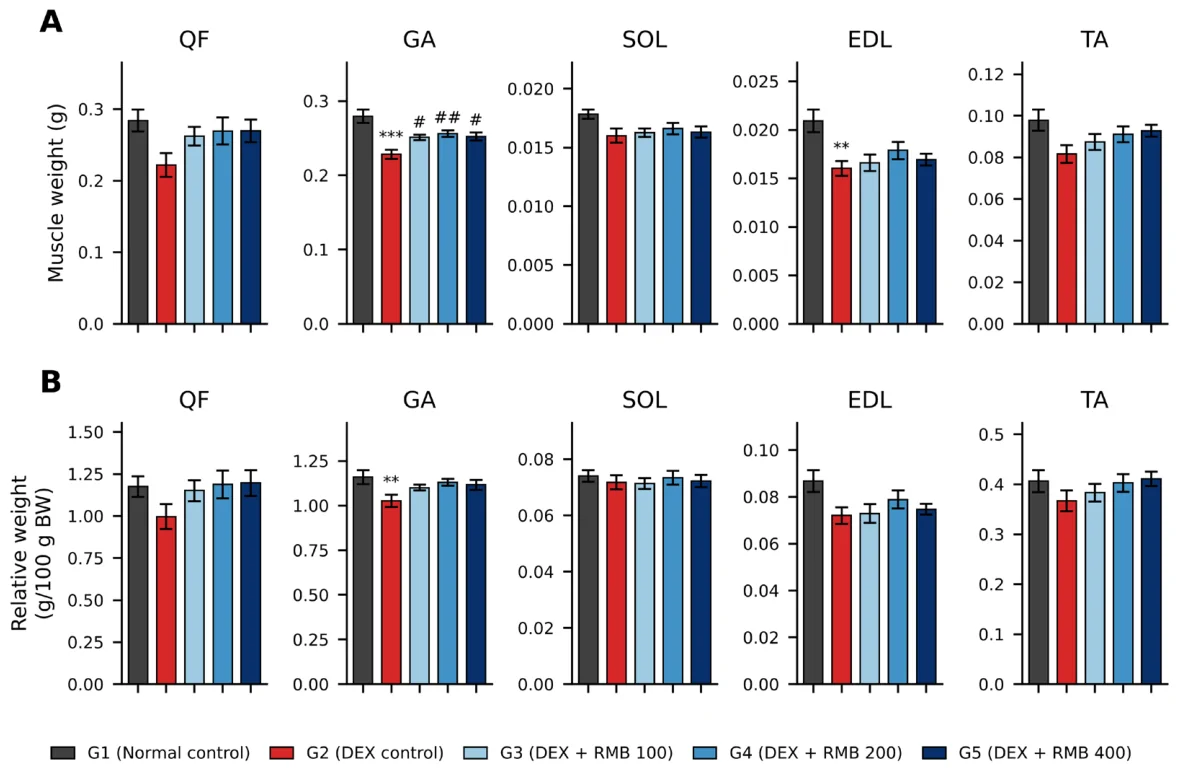
Effects of RMB on skeletal muscle weight. (**A**) Absolute muscle weight (g) and (**B**) relative muscle weight (g/100 g BW). QF, quadriceps femoris; GA, gastrocnemius; SOL, soleus; EDL, extensor digitorum longus; TA, tibialis anterior. Values are expressed as mean ± SEM (*n* = 10 per group). ** *p* < 0.01, *** *p* < 0.001 vs. G1; # *p* < 0.05, ## *p* < 0.01 vs. G2 (one-way ANOVA followed by Bonferroni-corrected pairwise comparisons). G1, normal control; G2, DEX control; G3–G5, DEX + RMB 100, 200, and 400 mg/kg.

As shown in Figure 3A, the absolute weights of the quadriceps femoris (QF), gastrocnemius (GA), soleus (SOL), extensor digitorum longus (EDL), and tibialis anterior (TA) tended to decrease in G2 compared with G1, and the decreases in GA and EDL weights were statistically significant. Compared with G2, GA weight was significantly increased in all RMB-treated groups. When normalized to body weight (g/100 g body weight [BW]), only the relative GA weight was significantly reduced in G2, and no significant differences were observed between G2 and the RMB-treated groups (Figure 3B).

### 2.6. Effects on Muscle Fiber Cross-Sectional Area of the Tibialis Anterior (TA)

**Figure 4:**
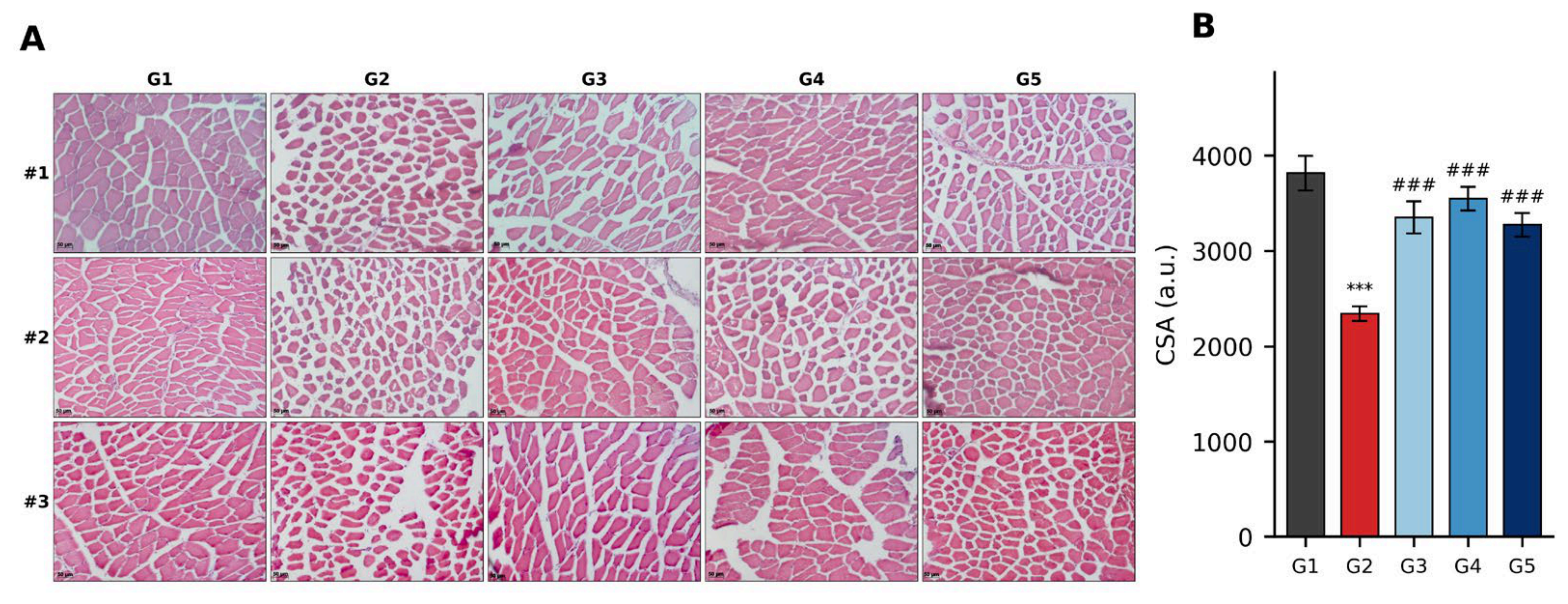
Effects of RMB on muscle fiber cross-sectional area (CSA) of the tibialis anterior (TA). (**A**) Representative H&E-stained transverse sections of the TA (×200; scale bar = 50 µm); #1–#3 indicate three different representative animals from each group. (**B**) Muscle fiber CSA. Values are expressed as mean ± SEM (*n* = 10 per group). *** *p* < 0.001 vs. G1; ### *p* < 0.001 vs. G2 (one-way ANOVA followed by Bonferroni-corrected pairwise comparisons). G1, normal control; G2, DEX control; G3–G5, DEX + RMB 100, 200, and 400 mg/kg.

Hematoxylin and eosin (H&E) staining of the TA revealed smaller muscle fibers in G2 than in G1, whereas larger fibers were observed in the RMB-treated groups (Figure 4A). Quantitatively, muscle fiber cross-sectional area (CSA) was significantly reduced in G2 (2345 ± 77 a.u.) compared with G1 (3816 ± 182 a.u.) and was significantly increased in all RMB-treated groups compared with G2 (Figure 4B).

### 2.7. Effects on Protein Levels in the Gastrocnemius (GA)

#### 2.7.1. Effects on Signaling Pathways Regulating Muscle Protein Synthesis and Degradation

**Figure 5:**
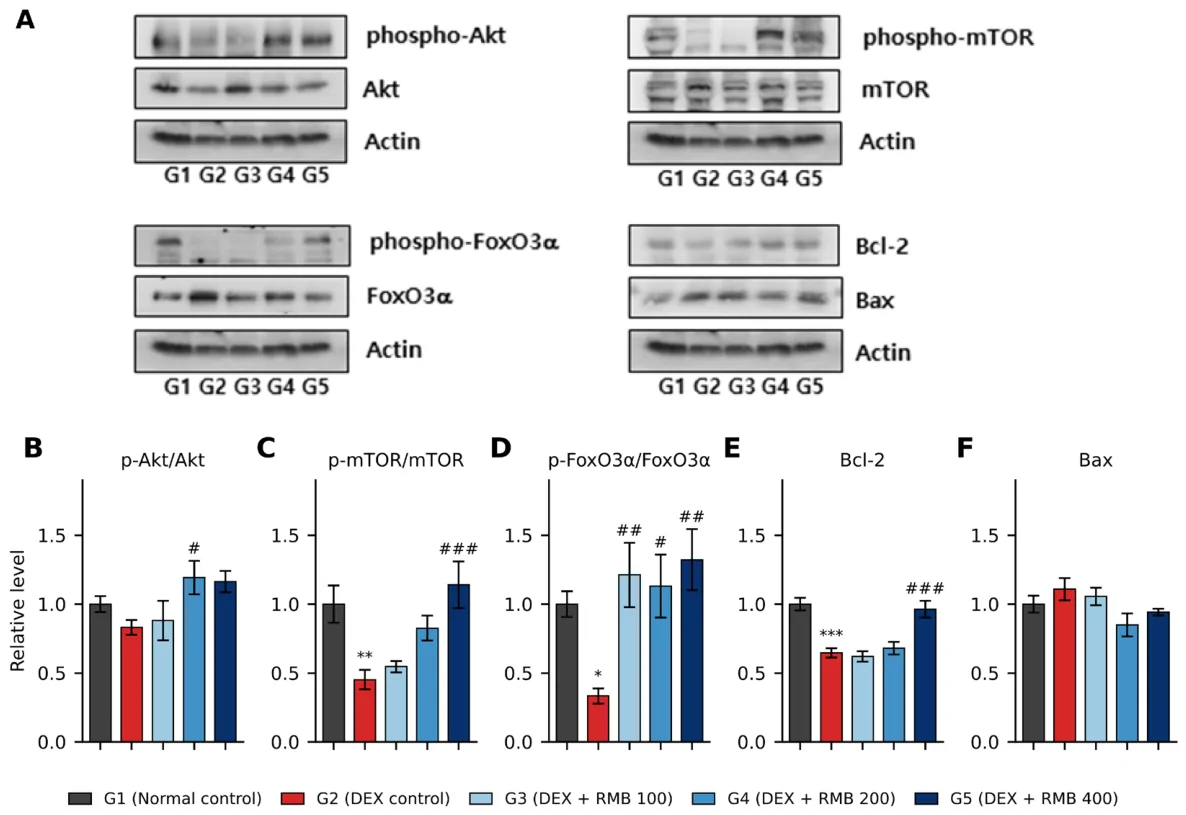
Effects of RMB on protein levels in the gastrocnemius (GA). (**A**) Representative Western blots of phospho-Akt, Akt, phospho-mTOR, mTOR, phospho-FoxO3α, FoxO3α, Bcl-2, Bax, and actin. All target proteins were detected on the same membrane; therefore, the same actin blot is shown as the loading control for each set of target proteins. (**B**) p-Akt/Akt, (**C**) p-mTOR/mTOR, and (**D**) p-FoxO3α/FoxO3α ratios, and relative (**E**) Bcl-2 and (**F**) Bax levels. Values are expressed as mean ± SEM (*n* = 12 for G1 and *n* = 10 for G2–G5). * *p* < 0.05, ** *p* < 0.01, *** *p* < 0.001 vs. G1; # *p* < 0.05, ## *p* < 0.01, ### *p* < 0.001 vs. G2 (one-way ANOVA followed by Bonferroni-corrected pairwise comparisons). G1, normal control; G2, DEX control; G3–G5, DEX + RMB 100, 200, and 400 mg/kg.

The levels of Akt, mTOR, and FoxO3α and their phosphorylated forms in the GA were analyzed by Western blotting (Figure 5).

Phospho-Akt levels were significantly decreased in the DEX control group (G2) compared to the normal control group (G1) and tended to recover in the RMB-treated groups (G3, G4, G5). Akt levels did not differ significantly between G2 and G1 or between G2 and the RMB-treated groups (Figure 5A). Evaluation of Akt activity through the phospho-Akt/Akt ratio revealed a decreasing tendency in the DEX control group (G2) compared to the normal control group (G1). Among the DEX-treated groups (G2, G3, G4, G5), this ratio was significantly increased in the 200 mg/kg BW RMB-treated group (G4) compared to the DEX control group (G2) (Figure 5B).

As shown in Figure 5A, phospho-mTOR levels were significantly decreased in the DEX control group (G2) compared to the normal control group (G1). Among the DEX-treated groups (G2, G3, G4, G5), phospho-mTOR levels tended to increase in the 200 and 400 mg/kg BW RMB-treated groups (G4, G5). There was no significant difference in mTOR levels between the normal control group (G1) and the DEX control group (G2). However, among the DEX-treated groups (G2, G3, G4, and G5), mTOR levels were significantly decreased in the 400 mg/kg BW RMB-treated group (G5) compared to the DEX control group (G2). Evaluation of mTOR activity through the phospho-mTOR/mTOR ratio revealed a significant decrease in the DEX control group (G2) compared to the normal control group (G1). This ratio was significantly increased in the 400 mg/kg BW RMB-treated group (G5) compared to the DEX control group (G2) (Figure 5C).

Phospho-FoxO3α levels significantly decreased in the DEX control group (G2) compared to the normal control group (G1), as shown in Figure 5A. Among the DEX-treated groups (G2, G3, G4, and G5), phospho-FoxO3α levels tended to increase in the 400 mg/kg BW RMB-treated group (G5). FoxO3α levels significantly increased in the DEX control group (G2) compared to the normal control group (G1), and among the DEX-treated groups (G2, G3, G4, and G5), FoxO3α levels significantly decreased in all RMB-treated groups (G3, G4, and G5) compared to the DEX control group (G2) (Figure 5A). The ratio of phospho-FoxO3α to FoxO3α significantly decreased in the DEX control group (G2) compared to the normal control group (G1) and significantly increased in all RMB-treated groups (G3, G4, and G5) (Figure 5D).

#### 2.7.2. Effects on the Levels of Apoptosis Regulatory Proteins

As shown in Figure 5A,E, Bcl-2 levels significantly decreased in the DEX control group (G2) compared to the normal control group (G1) but significantly increased in the 400 mg/kg BW RMB-treated group (G5) compared to the DEX control group (G2). Bax levels showed no significant differences among the groups (Figure 5A,F).

### 2.8. Effects on mRNA Expression in Muscle (Soleus Muscle, SOL)

#### 2.8.1. Effects on Gene Expression Related to Muscle Degradation and Formation

**Figure 6:**
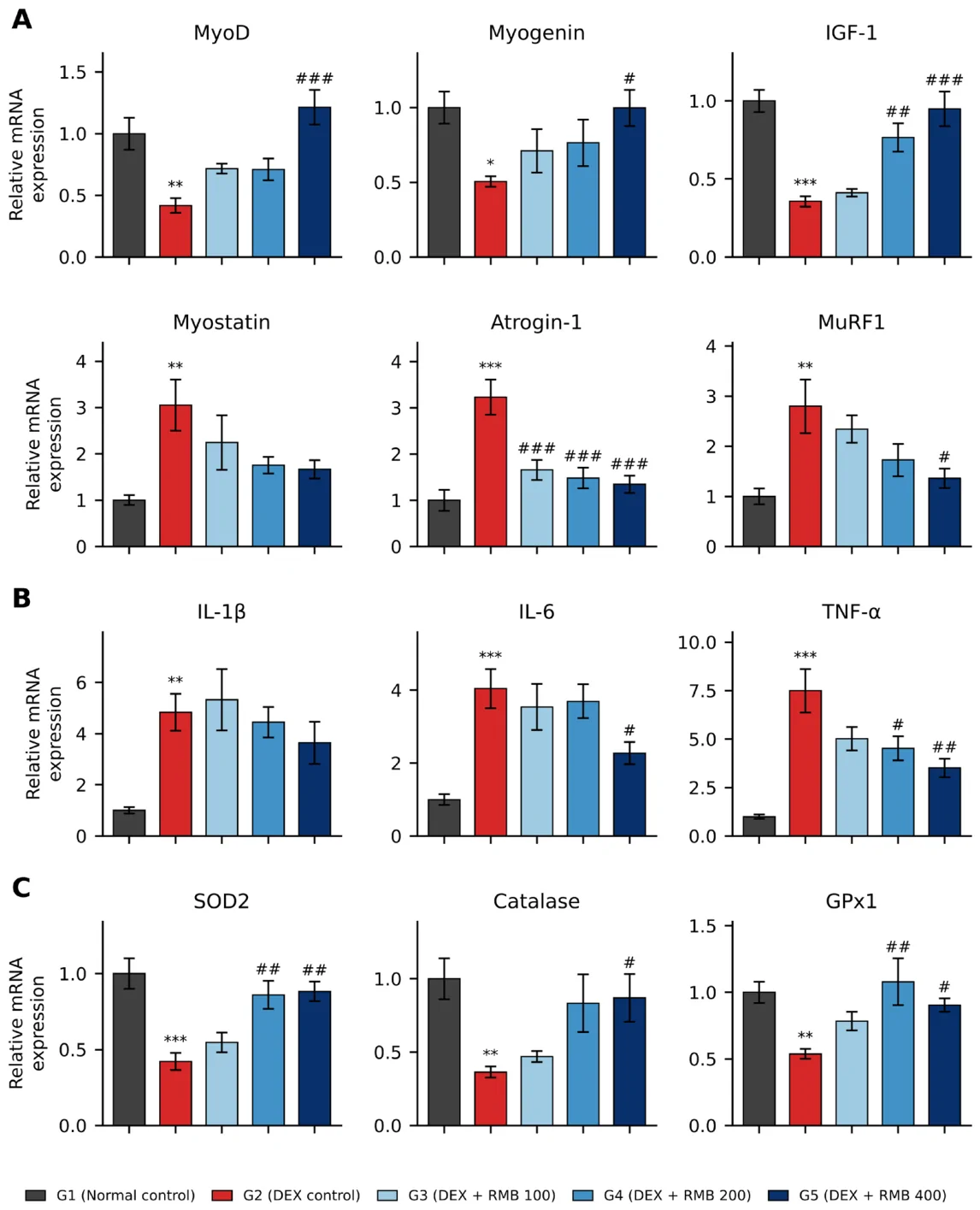
Effects of RMB on mRNA expression in the soleus (SOL). (**A**) Genes related to muscle formation and atrophy, (**B**) inflammation-related genes, and (**C**) antioxidant genes. Expression levels were normalized to glyceraldehyde-3-phosphate dehydrogenase (*GAPDH*) and expressed relative to G1. Values are expressed as mean ± SEM (*n* = 6 per group). * *p* < 0.05, ** *p* < 0.01, *** *p* < 0.001 vs. G1; # *p* < 0.05, ## *p* < 0.01, ### *p* < 0.001 vs. G2 (one-way ANOVA followed by Bonferroni-corrected pairwise comparisons). G1, normal control; G2, DEX control; G3–G5, DEX + RMB 100, 200, and 400 mg/kg.

The mRNA expression of muscle formation-related genes (myogenic differentiation 1 [*MyoD*], *Myogenin*, and *IGF-1*) and muscle atrophy-related genes (*Myostatin*, *Atrogin-1*, and *MuRF1*) in the SOL is shown in Figure 6A. The mRNA expression levels of muscle formation-related genes *MyoD*, *Myogenin*, and *IGF-1* were significantly decreased in the DEX control group (G2) compared to the normal control group (G1). *MyoD* mRNA expression, which was significantly reduced in the DEX control group (G2), was significantly increased in the 400 mg/kg BW RMB-treated group (G5). *Myogenin* mRNA expression, also reduced in G2, significantly increased in the 400 mg/kg BW RMB-treated group (G5). *IGF-1* mRNA expression, decreased in G2, significantly increased in both the 200 mg/kg BW RMB-treated group (G4) and the 400 mg/kg BW RMB-treated group (G5).

The mRNA expression of muscle atrophy-related genes *Myostatin*, *Atrogin-1*, and *MuRF1* was significantly elevated in the DEX control group (G2) compared to the normal control group (G1). Among the DEX-treated groups (G2, G3, G4, and G5), *Myostatin* mRNA expression tended to decrease in a dose-dependent manner in the RMB-treated groups. *Atrogin-1* mRNA expression was significantly decreased in all RMB-treated groups (G3, G4, and G5) relative to G2. *MuRF1* mRNA expression showed a significant decrease only in the 400 mg/kg BW RMB-treated group (G5) compared to G2 (Figure 6A).

#### 2.8.2. Effects on the Expression of Inflammation-Related Genes

The mRNA levels of inflammation-related genes interleukin-1β (*IL-1β*), *IL-6*, and *TNF-α* were examined in the soleus muscle, and the results are summarized in Figure 6B. As shown in Figure 6B, the mRNA expression of *IL-1β*, *IL-6*, and *TNF-α* in the soleus muscle was significantly increased in the DEX control group (G2) compared to the normal control group (G1). Among the DEX-treated groups (G2, G3, G4, and G5), *IL-6* mRNA expression was significantly decreased in the 400 mg/kg BW RMB-treated group (G5) compared to the DEX control group (G2), while *TNF-α* mRNA expression was significantly reduced in the 200 mg/kg BW RMB-treated group (G4) and the 400 mg/kg BW RMB-treated group (G5). *IL-1β* mRNA expression tended to decrease in the 200 and 400 mg/kg BW RMB-treated groups (G4 and G5) (Figure 6B).

#### 2.8.3. Effects on the Expression of Genes Related to Antioxidants

The mRNA levels of the antioxidant genes superoxide dismutase 2 (*SOD2*), *catalase*, and glutathione peroxidase 1 (*GPx1*) were examined in the soleus muscle. As shown in Figure 6C, the expression of *SOD2*, *catalase*, and *GPx1* mRNA was significantly decreased in the DEX control group (G2) compared to the normal control group (G1). Among the DEX-treated groups (G2, G3, G4, and G5), *SOD2* mRNA expression was significantly increased in the 200 mg/kg BW RMB-treated group (G4) and the 400 mg/kg BW RMB-treated group (G5), and *catalase* mRNA expression was significantly increased in G5 compared to the DEX control group (G2). *GPx1* mRNA expression was significantly elevated in G4 and G5 compared to the DEX control group (G2) (Figure 6C).

### 2.9. Effects on Serum Glutathione Content and Antioxidant Enzyme Activity

**Figure 7:**
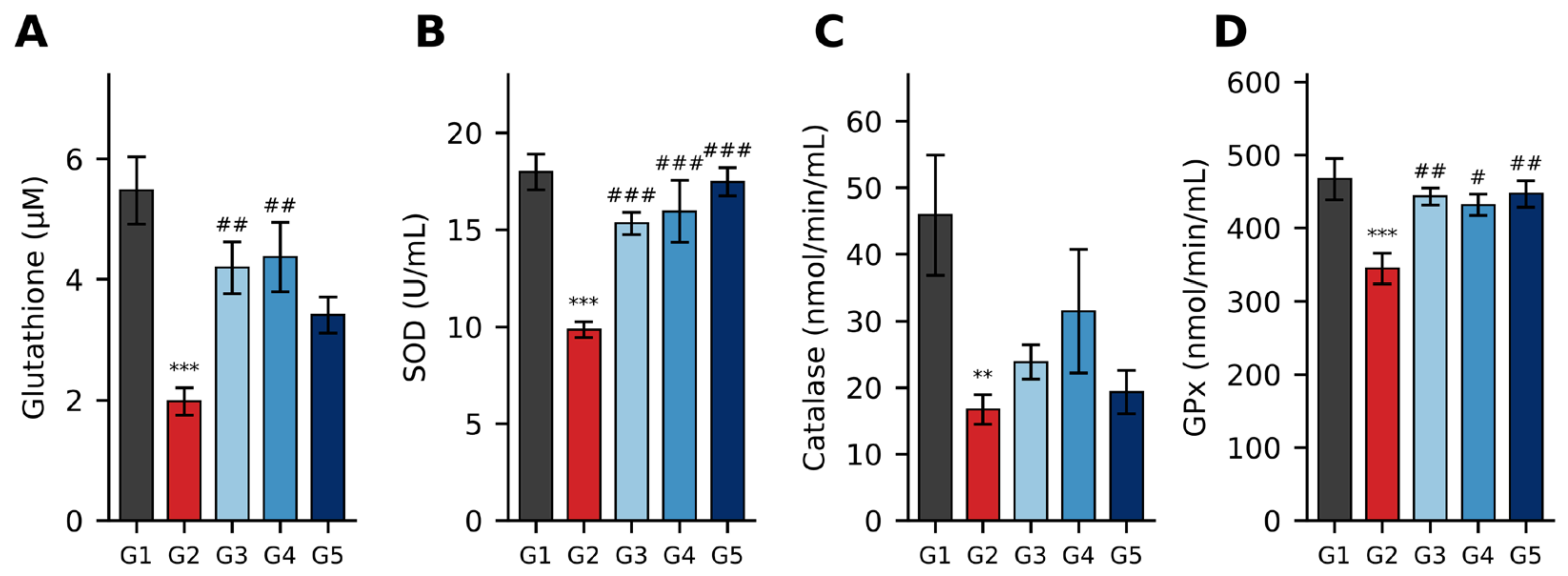
Effects of RMB on serum antioxidant status. (**A**) Glutathione content, (**B**) SOD activity, (**C**) catalase activity, and (**D**) GPx activity. Values are expressed as mean ± SEM (*n* = 10 per group). ** *p* < 0.01, *** *p* < 0.001 vs. G1; # *p* < 0.05, ## *p* < 0.01, ### *p* < 0.001 vs. G2 (one-way ANOVA followed by Bonferroni-corrected pairwise comparisons). G1, normal control; G2, DEX control; G3–G5, DEX + RMB 100, 200, and 400 mg/kg.

Serum glutathione content and the activities of superoxide dismutase (SOD), catalase, and glutathione peroxidase (GPx) are shown in Figure 7. Serum glutathione levels were significantly decreased in the DEX control group (G2) compared to the normal control group (G1). Among the DEX-treated groups (G2, G3, G4, and G5), the 100 and 200 mg/kg BW RMB-treated groups (G3 and G4) showed a significant increase in serum glutathione levels compared to the DEX control group (G2), and an increasing trend was also observed in the 400 mg/kg BW RMB-treated group (G5) (Figure 7A). Serum SOD and GPx activities were significantly reduced in G2 compared to G1 but were significantly increased in all RMB-treated groups (G3, G4, and G5) compared to G2 (Figure 7B,D). Serum catalase activity was significantly decreased in G2 compared to G1 and tended to increase in the RMB-treated groups (G3, G4, and G5) compared to the DEX control group (G2) (Figure 7C).

## 3. Discussion

In the present study, oral administration of RMB attenuated DEX-induced muscle atrophy at the organism, organ, cellular, and molecular levels, encompassing both functional and structural outcomes. RMB improved endurance at 200 and 400 mg/kg, preserved muscle fiber size and gastrocnemius mass, partially restored Akt/mTOR/FoxO3α signaling, suppressed muscle-specific atrophy genes, and attenuated inflammatory, oxidative, and pro-apoptotic changes. These findings extend our previous in vitro observations [43] to an in vivo model that is widely used to reproduce key features of muscle wasting, including loss of muscle mass and function and altered signaling of muscle protein turnover [69].

DEX reduced body weight from week 3, consistent with the catabolic effects of glucocorticoids [69], and RMB did not restore overall body weight; thus, the benefits of RMB were specific to muscle-related outcomes rather than reflecting general recovery of body weight. No mortality or adverse clinical signs were observed in any group. The improvement in exercise performance at 200 and 400 mg/kg is consistent with previous studies in DEX models in which functional recovery was accompanied by restoration of Akt/mTOR signaling [70,71]. Grip strength also tended to recover at 200 and 400 mg/kg (approximately 20 g higher than in the DEX control group), in line with the grip-strength recovery reported for other flavonoid-containing preparations in DEX models [71,72]. Notably, CSA and GA weight were significantly restored, even at 100 mg/kg, whereas grip strength showed only a trend toward recovery, suggesting that structural recovery of muscle fibers may precede measurable recovery of muscle strength within the 4-week treatment period; studies with longer treatment periods and assessments of neuromuscular function are needed to test this possibility. The trends toward normalization of body fat and lean body percentages did not reach statistical significance, possibly because the 4-week treatment period was too short to detect changes in whole-body composition.

The responses to DEX and RMB also differed among muscles. DEX significantly reduced the absolute weights of the fast-twitch-dominant GA and EDL and the relative weight of the GA, whereas the weights of the QF, TA, and slow-twitch-dominant SOL were not significantly affected, suggesting that the GA may be particularly susceptible to DEX-induced atrophy. Consistently, the effect of RMB on muscle weight was observed in the GA, in which absolute weight was significantly increased at all doses.

At the molecular level, the most prominent finding was the restoration of the Akt/mTOR/FoxO3α axis. The IGF-1/phosphoinositide 3-kinase (PI3K)/Akt/mTOR pathway is a central regulator of muscle protein synthesis, and its suppression by glucocorticoids is a major mechanism of DEX-induced muscle atrophy [73]. RMB increased the p-Akt/Akt ratio at 200 mg/kg and the p-mTOR/mTOR ratio at 400 mg/kg, whereas the p-FoxO3α/FoxO3α ratio was restored at all doses, including 100 mg/kg, indicating that FoxO3α was among the most responsive targets of RMB. Because Akt-mediated phosphorylation of FoxO3α suppresses its transcriptional activity, this change was consistent with the downregulation of its target genes *Atrogin-1* (at all doses) and *MuRF1* (at 400 mg/kg). *Myostatin*, a member of the transforming growth factor-β (TGF-β) family that suppresses Akt/mTOR/p70 ribosomal S6 kinase (p70S6K) signaling, showed a dose-dependent decreasing trend with RMB treatment, whereas myogenic and anabolic markers *MyoD* and *Myogenin* (at 400 mg/kg) and *IGF-1* (at 200 and 400 mg/kg) were restored, in line with reports linking Akt/mTOR activation to *MyoD* and *Myogenin* expression [74,75]. Similar restoration of Akt/mTOR/FoxO3α signaling has been reported for other flavonoid-containing natural extracts [70,71,76,77] and in our previous in vitro and in vivo studies of *Ulmus macrocarpa* and *Alnus japonica* extracts [39,40,41,42].

RMB also attenuated the inflammatory, oxidative, and apoptotic changes that contribute to muscle atrophy. RMB reduced intramuscular *TNF-α* and *IL-6*, cytokines that promote muscle protein degradation through nuclear factor-κB (NF-κB)-mediated ubiquitin-proteasome signaling, consistent with the cytokine-modulatory activities of taxifolin-type flavonoids [65] and with our previous findings [40,42]. RMB also restored antioxidant defense in both muscle (*SOD2*, *catalase*, and *GPx1* mRNA) and serum (glutathione content and SOD and GPx activities). Because SOD2 is the mitochondrial isoform of superoxide dismutase, its restoration indirectly suggests improved mitochondrial redox status, although mitochondrial function was not directly assessed. For apoptosis, the present study focused on Bcl-2 and Bax, the key anti- and pro-apoptotic regulators, to verify in muscle tissue the anti-apoptotic effects observed in our previous in vitro study, in which RMB and its active flavonoids increased Bcl-2 and suppressed cleaved caspase-3 and poly(ADP-ribose) polymerase (PARP) [43]. RMB restored Bcl-2 at 400 mg/kg, consistent with these in vitro findings and with our previous studies [39,40,41,42], although Bax levels were not significantly altered by DEX or RMB. Rather than establishing a direct causal sequence, these concurrent changes suggest an integrated cytoprotective environment that may contribute to the preservation of muscle protein homeostasis [40,42,65].

Some indicators did not show a clear dose-dependent pattern: muscle fiber CSA and GA weight were highest at 200 mg/kg, serum glutathione was significantly increased at 100 and 200 mg/kg but not at 400 mg/kg, the p-Akt/Akt ratio was significantly increased only at 200 mg/kg, and Bcl-2 recovered only at 400 mg/kg. Several factors may explain these patterns. First, different indicators were measured in different muscles (TA for CSA, GA for proteins, and SOL for genes), and these muscles may respond differently to DEX. Second, the intestinal absorption of flavonoid glycosides such as Tax-G may reach its limit at higher doses, so that a further increase in dose does not necessarily increase the effect. Third, polyphenols often show their greatest effect at intermediate doses, particularly on antioxidant-related indicators. In contrast, the main functional outcomes (exercise time and exercise capacity) and the key muscle-breakdown markers (*Atrogin-1*, *MuRF1*, and *Myostatin*) showed consistent dose-dependent trends.

The phytochemical profile of RMB provides a plausible basis for these biological effects. Thin-layer chromatography (TLC) and high-performance liquid chromatography (HPLC) analyses in our previous study [43] identified taxifolin-3-O-arabinopyranoside (Tax-G; 107.47 ± 0.45 µg/mL) as a major flavonoid marker of RMB, consistent with our chemotaxonomic studies of the genus *Rhododendron*, which established Tax-G as a characteristic marker compound of *R. mucronulatum* [62,63,64]. Tax-G belongs to the taxifolin (dihydroquercetin) class of flavonoids, which has been associated with antioxidant, anti-inflammatory, and cytoprotective activities [65]. In our previous in vitro study, Tax-G showed lower cytotoxicity than taxifolin aglycone (Tax-A) while suppressing *Atrogin-1*, *MuRF1*, and FoxO3α and increasing *MyoD*, *Myogenin*, Akt, and mTOR in DEX-treated C2C12 cells [43]. The consistency between these cellular findings and the present in vivo results suggests that Tax-G may contribute to the activity of RMB. Because RMB was evaluated as a standardized extract with Tax-G as its marker compound, the effects observed in this study are attributed to RMB, and the in vivo contribution of Tax-G as an isolated compound will be examined in future studies.

With regard to the development of RMB as a functional ingredient, the same batch of RMB used in this study showed no treatment-related adverse effects in a toxicological battery conducted in compliance with Good Laboratory Practice (GLP) in accordance with the MFDS GLP regulations and the OECD Test Guidelines. It was classified as Category 5/unclassified under the Globally Harmonized System of Classification and Labelling of Chemicals (GHS) in a single-dose oral toxicity study in rats (2000 mg/kg), yielded a no-observed-adverse-effect level (NOAEL) of 1000 mg/kg/day (the highest dose tested) in both sexes in a 90-day repeated-dose oral toxicity study in Sprague-Dawley rats, and was negative in bacterial reverse mutation, in vitro chromosomal aberration, and in vivo micronucleus assays.

Several limitations should be considered. First, the DEX-induced model represents glucocorticoid-induced secondary muscle atrophy rather than primary age-related sarcopenia. Wang et al. (2023) reported similarities between DEX-treated young mice and naturally aged mice in muscle mass, muscle fiber size, and expression of genes associated with protein degradation [69], supporting the utility of the DEX model as a surrogate for selected features of sarcopenia. Nevertheless, the relevance of RMB to primary age-related sarcopenia requires confirmation in aged-animal models and ultimately in clinical studies. Second, only male mice were used to avoid variability arising from estrous cycle-dependent fluctuations in estrogen, which can influence glucocorticoid sensitivity and muscle protein turnover, and to maintain consistency with our previous studies using the same model [40,42]; therefore, sex differences in the response to RMB remain to be determined. Third, autophagy, mitochondrial function, and muscle fiber-type transitions were not directly evaluated, although these processes are important components of muscle aging and atrophy and may also be influenced by RMB. Finally, although RMB showed no adverse effects in GLP-compliant preclinical toxicity studies, its efficacy and safety in humans remain to be confirmed in clinical trials. Future studies addressing these limitations will be important for defining the efficacy, mechanism, and translational potential of RMB for the management of age-related muscle loss.

## 4. Materials and Methods

### 4.1. Plant Material and Experimental Samples

The *R. mucronulatum* branch extract (RMB) was identical to that used in our previous in vitro study [43]. Briefly, *R. mucronulatum* branches were purchased from the Seoul Yakryeong Medicine Market (Seoul, Korea) and certified by Professor Choi (Department of Forest Biomaterials Engineering, Kangwon National University). RMB was manufactured on a pilot scale under Good Manufacturing Practice (GMP) conditions by Danjoungbio Co., Ltd. (Wonju, Korea) (Lot No. DJTH-05572), and stored at the Department of Forest Biomaterials Engineering, Kangwon National University. The same batch of RMB as that used in our previous in vitro study [43] was applied directly to the present in vivo experiments.

### 4.2. Phytochemical Analysis (TLC and HPLC)

Qualitative TLC analysis and quantitative HPLC analysis of RMB were performed as described in our previous in vitro study [43]. Briefly, TLC was conducted on silica gel plates (Silica gel 60 F254; Sigma-Aldrich, St. Louis, MO, USA) using a mobile phase of chloroform:methanol:water (70:30:4, *v*/*v*/*v*) and visualized under UV light (254 nm) and with spray reagents (10% H_2_SO_4_, ρ-anisaldehyde-H_2_SO_4_, FeCl_3_). HPLC was performed using a Waters 2695 system (Waters, Milford, MA, USA) with an OptimaPak C18 column (250 × 4.6 mm, 5 µm; Chiral Technology Korea Co., Ltd., Daejeon, Korea) at a detection wavelength of 280 nm.

### 4.3. Ethical Statement and Animals

All animal experiments were conducted in accordance with the regulations for the use and care of experimental animals established by the Ministry of Food and Drug Safety (MFDS), and approval for the experimental procedures was obtained from the Institutional Animal Care and Use Committee (IACUC) of Hallym University, which is accredited by the National Agricultural Products Quality Management Service under the Ministry of Agriculture, Food and Rural Affairs of Korea (approval no.: Hallym 2023-52).

Male C57BL/6 mice, 8 weeks old (young adult) and specific pathogen-free (SPF), were purchased from Dooyeol Biotech Co., Ltd. (Seoul, Korea). After a one-week quarantine and acclimatization period, healthy animals without weight loss were selected for the experiment. The experimental animals were housed four per cage to avoid crowding stress under controlled conditions at a temperature of 23 ± 3 °C, a relative humidity of 50 ± 10%, a ventilation rate of 10–15 air changes per hour, a 12-h light/dark cycle (08:00–20:00), and an illumination intensity of 150–300 lux. Throughout the study, the animals were provided with sterilized solid rodent feed (Purina Rodent Chow, product code 38057; Cargill Agri Purina Co., Ltd., Seoul, Korea) and sterilized water ad libitum, and cages were changed regularly to maintain a clean environment.

### 4.4. Experimental Design and Treatment

**Figure 8:**
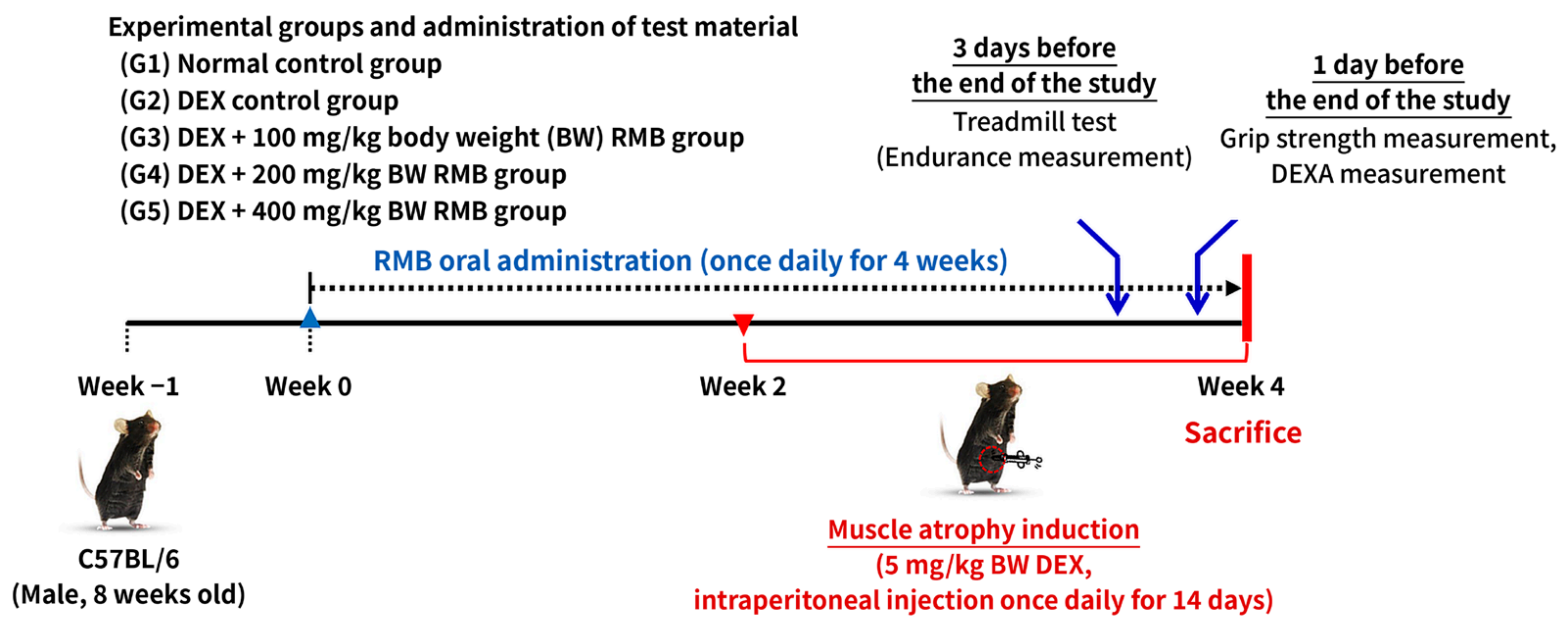
Experimental design. After a 1-week acclimation period, RMB (100, 200, or 400 mg/kg) was administered orally once daily for 4 weeks, and DEX (5 mg/kg) was injected intraperitoneally once daily during the final 2 weeks. The treadmill test was performed 3 days before the end of the experiment, and the grip strength test and dual-energy X-ray absorptiometry (DEXA) were performed 1 day before the end, followed by sacrifice and collection of serum and muscle tissues. Body weight was measured weekly.

Male C57BL/6 mice were used in this study because C57BL/6 is one of the most widely used strains in DEX-induced muscle atrophy models and because the use of a single sex avoids variability associated with the estrous cycle, as fluctuations in estrogen can influence glucocorticoid responsiveness and muscle protein metabolism. This choice also maintained consistency with our previous in vivo studies using the same model [40,42], allowing direct comparison of the results. Male C57BL/6 mice were acclimated for one week, after which healthy animals were selected and randomly divided into five groups according to a random block design: (G1) normal control group, (G2) DEX control group, (G3) DEX + RMB 100 mg/kg-treated group, (G4) DEX + RMB 200 mg/kg-treated group, and (G5) DEX + RMB 400 mg/kg-treated group, as shown in Table 1 and Figure 8. The RMB was dissolved in drinking water and orally administered once daily at a fixed time for 4 weeks. After 2 weeks of RMB administration, muscle atrophy was induced by intraperitoneal injection of dexamethasone (DEX; Cat. No. D4902; Sigma-Aldrich) at 5 mg/kg BW once daily for 14 days. Body weights were measured once weekly at a fixed time starting from the first day of RMB administration. The mean body weight at the start of RMB administration was 20.8 ± 0.2 g.

### 4.5. Measurement of Exercise Capacity

Endurance exercise capacity was assessed using a four-lane small-animal treadmill (MZ-2021V152, HUAYON Biotech Co., Ltd., Shenzhen, China) three days before the end of the experiment, according to a published method [78]. To evaluate intrinsic endurance without learning or training effects, no pre-training or acclimatization session was performed before the test day. Because of the four-lane configuration, four mice from each group were tested in rotation (e.g., four mice from G1, followed by four from G2 and four from G3) to minimize bias related to the time of testing. The exercise started at a 10° incline and a speed of 10 m/min for 5 min, after which the speed was increased by 1 m/min every minute up to a maximum of 25 m/min. Exhaustion was defined as the point at which the animal was unable to keep running and remained at the rear of the treadmill for more than 10 s, and the time to exhaustion was recorded. Exercise capacity, which represents the total physical work performed against gravity during uphill running, was calculated as in our previous studies [40,42] using the formula of Brooks and White [79]: Exercise capacity (J, kg × m^2^ × s^−2^) = body weight (kg) × speed (m/s) × time (s) × grade × 9.8 m/s^2^. Because DEX reduced body weight, exercise capacity was reported together with time to exhaustion to account for differences in body weight among groups.

### 4.6. Measurement of Grip Strength

Grip strength was measured one day before the end of the experiment using a small-animal grip strength meter (BIO-GS3; Bioseb, Vitrolles, France), as described previously [80]. To evaluate intrinsic muscle strength without learning effects, the animals were not trained on the apparatus before the test day, and all measurements were completed between 09:00 and 12:00 to maintain consistent testing conditions. Each animal was guided to grasp the T-bar with both forepaws and was then pulled by the tail at a constant speed of 2 cm/s until it released both forepaws, and the force (g) at the moment of release was recorded. Five measurements were obtained for each animal, and the mean value was used for analysis.

### 4.7. Measurement of Lean Body Percentage and Fat Percentage

One day before the end of the experiment, the animals were anesthetized, and body composition was measured using dual-energy X-ray absorptiometry (DEXA, PIXImus™, GE Lunar, Madison, WI, USA) to evaluate lean body mass percentage and body fat percentage.

### 4.8. Blood and Tissue Collection

To minimize pain and distress, the animals were anesthetized before blood collection and sacrifice by intraperitoneal injection of 2,2,2-tribromoethanol (Avertin; Cat. No. T48402; Sigma-Aldrich) diluted with tert-amyl alcohol (Cat. No. 240486; Sigma-Aldrich). Blood was collected via orbital puncture and transferred to serum separator tubes (BD Microtainer^®^ SST™, Cat. No. 365967; Becton, Dickinson and Company, Franklin Lakes, NJ, USA). The blood samples were left at room temperature for 30 min and then centrifuged at 5000 rpm for 10 min to separate the serum, which was stored at −70 °C until analysis. After blood collection, the animals were sacrificed, and skeletal muscles [quadriceps femoris (QF), gastrocnemius (GA), soleus (SOL), extensor digitorum longus (EDL), and tibialis anterior (TA)] were harvested. The muscles were rinsed with cold saline, excess moisture was removed using filter paper, and their weights were measured. Total RNA was extracted from the soleus (SOL) for real-time PCR analysis. A portion of the gastrocnemius (GA) muscle was used for protein extraction followed by Western blot analysis. Another portion of the tibialis anterior (TA) muscle was fixed in 4% paraformaldehyde (PFA; Cat. No. PC2031-100-00; Biosesang, Yongin, Korea), frozen, and sectioned for histological staining. The remaining tissues were stored at −70 °C.

### 4.9. Measurement of Antioxidant Enzyme Activity and Glutathione Content in Serum

The activities of superoxide dismutase (SOD), catalase, and glutathione peroxidase (GPx) and the glutathione content in serum were measured using the Superoxide Dismutase Assay Kit (Cat. No. 706002), Catalase Assay Kit (Cat. No. 707002), Glutathione Peroxidase Assay Kit (Cat. No. 703102), and Glutathione Assay Kit (Cat. No. 703002) (Cayman Chemical, Ann Arbor, MI, USA), respectively, according to the manufacturer’s instructions. Absorbance was measured using a SpectraMax microplate reader (Molecular Devices, San Jose, CA, USA). SOD activity was determined by measuring the extent to which SOD in the sample inhibited the formation of formazan dye from a tetrazolium salt reduced by superoxide radicals generated by xanthine oxidase; absorbance was measured at 450 nm, and activity was calculated from the inhibition rate. Catalase activity was determined on the basis of the reaction of catalase with methanol in the presence of H_2_O_2_ to produce formaldehyde, which was quantified with Purpald as the chromogen at 540 nm. GPx activity was measured by a coupled reaction in which oxidized glutathione (GSSG) produced by GPx was reduced by glutathione reductase and NADPH, and the oxidation of NADPH to NADP+ was monitored as the rate of decrease in absorbance at 340 nm [81]. Total glutathione (reduced glutathione [GSH] and GSSG) content was determined by an enzymatic recycling method, in which the sulfhydryl group of GSH reacts with 5,5′-dithiobis(2-nitrobenzoic acid) (DTNB) to produce yellow 5-thio-2-nitrobenzoic acid (TNB), and the formed GSSG is recycled to GSH by glutathione reductase and NADPH; the rate of TNB formation was measured at 405–414 nm [82].

### 4.10. Hematoxylin and Eosin Staining

The tibialis anterior (TA) muscle was fixed in 4% PFA in phosphate-buffered saline (PBS) for 24 h at room temperature, then cryoprotected in 30% sucrose at 4 °C until the tissue sank to the bottom of the container. To evaluate the muscle fiber cross-sectional area (CSA), the muscle was cut in half transversely, perpendicular to its longitudinal axis, embedded in Tissue-Tek^®^ O.C.T.™ Compound (Cat. No. 4583; Sakura Finetek, Torrance, CA, USA), and frozen. Transverse cryosections (6 µm thick) were prepared at −25 °C using a cryostat (MICROM HM 520; Thermo Fisher Scientific, Waltham, MA, USA). Sections from all animals (*n* = 10 per group) were air-dried at room temperature, washed five times with PBS for 3 min each, and stained with Accustain^®^ Hematoxylin and Eosin Stains (Cat. Nos. H3136 and E4009; Sigma-Aldrich) according to the manufacturer’s instructions. For each animal, three fields showing uniformly cross-sectioned fibers were randomly selected and photographed at ×200 magnification using a light microscope (Axio Imager.M1; Carl Zeiss, Oberkochen, Germany) equipped with an AxioCam digital camera (Carl Zeiss). The most representative image from each animal was selected, and CSA was quantified using ImageJ software (version 1.54; National Institutes of Health, Bethesda, MD, USA).

### 4.11. Western Blot Analysis

To analyze protein levels in muscle tissue (gastrocnemius, GA), lysis buffer (20 mmol/L Hepes, pH 7.5; 150 mmol/L NaCl; 1% Triton X-100; 1 mmol/L EDTA; 1 mmol/L EGTA; 100 mmol/L NaF; 10 mmol/L sodium pyrophosphate; 1 mmol/L Na_3_VO_4_; 20 µg/mL aprotinin; 10 µg/mL antipain; 10 µg/mL leupeptin; 80 µg/mL benzamidine HCl; 0.2 mmol/L PMSF) was added, and the tissue was homogenized using a homogenizer. The homogenate was centrifuged at 12,000 rpm for 10 min, and the supernatant was collected to obtain muscle tissue lysates. Protein concentration in the lysates was measured using a bicinchoninic acid (BCA) protein assay kit (Cat. No. 23225; Thermo Scientific, Waltham, MA, USA).

Fifty micrograms of protein were separated by 10% sodium dodecyl sulfate polyacrylamide gel electrophoresis (SDS-PAGE) and transferred onto a polyvinylidene difluoride membrane (Immobilon^®^-P, 0.45 µm pore size, Cat. No. IPVH00010; Merck Millipore, Burlington, MA, USA). The membrane was blocked with 5% skim milk in Tris-buffered saline containing Tween 20 (TBST; 20 mmol/L Tris·HCl, pH 7.5; 150 mmol/L NaCl; 0.1% Tween 20) for 1 h, then incubated with specific primary antibodies (Cell Signaling Technology, Danvers, MA, USA; diluted 1:1000 in 5% bovine serum albumin (BSA) in TBST) either at 4 °C for 16 h or at room temperature for 1 h with gentle agitation. Information on the antibodies used is shown in Table 2. Next, the membrane was incubated with horseradish peroxidase (HRP)-linked anti-rabbit IgG (Cat. No. 7074S) or HRP-linked anti-mouse IgG (Cat. No. 7076S) (Cell Signaling Technology; diluted 1:5000 in 5% nonfat dry milk in TBST) for 1 h. Protein bands were visualized using enhanced chemiluminescence with Luminata™ Forte Western HRP Substrate (Cat. No. WBLUF0500; Millipore). Protein levels were quantified using the ImageQuant™ LAS 500 imaging system (GE Healthcare Bio-Sciences AB, Uppsala, Sweden).

### 4.12. Real-Time Polymerase Chain Reaction (Real-Time PCR)

Total RNA was extracted from the soleus muscle (SOL) collected at the end of the experiment using TRIzol^®^ Reagent (Cat. No. 15596018; Thermo Fisher Scientific). RNA quantification was performed using a micro-volume spectrophotometer (BioSpec-nano, Shimadzu, Kyoto, Japan), and RNA samples with an OD260/280 ratio greater than 1.8 were used for further analysis. Complementary DNA (cDNA) was synthesized from 2 μg of total RNA using the HyperScript™ RT master mix kit (Cat. No. 601710; GeneAll Biotechnology, Seoul, Korea). Real-time PCR was carried out using the Rotor-Gene 3000 PCR system (Corbett Research, Mortlake, NSW, Australia) and the QuantiNova™ SYBR Green PCR kit (Cat. No. 208056; Qiagen, Hilden, Germany). Primer information is presented in Table 3. Quantitative analysis of gene expression was performed using the Rotor-Gene 6000 Series System Software (version 6; Corbett Research), and relative gene expression levels were normalized to *GAPDH* and calculated using the 2^−ΔΔCt^ method [83].

### 4.13. Statistical Analysis

Data are presented as the mean ± SEM. Statistical analyses were performed using IBM SPSS Statistics (version 32.0; IBM Corp., Armonk, NY, USA). The normality of the data distribution within each group was assessed using the Shapiro–Wilk test. Of the 240 group-wise tests (48 parameters × 5 groups), 215 (89.6%) showed no significant deviation from normality (*p* > 0.05); the remaining deviations were mild (all *p* > 0.004) and sporadic and were not confined to any particular group or parameter (Table S1). Therefore, differences among the five groups were evaluated by one-way analysis of variance (ANOVA). When the one-way ANOVA was significant, predefined pairwise comparisons were performed with Bonferroni correction for four comparisons: the normal control group (G1) was compared with the DEX control group (G2) to confirm the induction of muscle atrophy, and each RMB-treated group (G3–G5) was compared with G2 to evaluate the effects of RMB. For the 19 parameters in which at least one group deviated from normality, the results were additionally confirmed using the non-parametric Kruskal–Wallis test, which yielded conclusions consistent with those of the one-way ANOVA for all parameters (Table S1). Statistical significance was set at *p* < 0.05.

## 5. Conclusions

In conclusion, oral administration of *R. mucronulatum* branch extract (RMB) significantly attenuated dexamethasone (DEX)-induced muscle atrophy in young adult male C57BL/6 mice. RMB improved exercise performance; preserved muscle fiber cross-sectional area and gastrocnemius weight; partially restored Akt/mTOR signaling and increased FoxO3α phosphorylation; suppressed muscle catabolic regulators *Atrogin-1* and *MuRF1*; and enhanced myogenic and anabolic markers including *MyoD*, *Myogenin*, and *IGF-1*. RMB also attenuated intramuscular inflammatory signaling and restored antioxidant defense, while the increase in Bcl-2 was consistent with a shift toward an anti-apoptotic state. Mechanistically, these findings suggest that RMB preserves skeletal muscle structure and function primarily by partially restoring Akt/mTOR signaling and increasing FoxO3α phosphorylation, accompanied by downregulation of *Atrogin-1* and *MuRF1* and upregulation of *IGF-1*, *MyoD*, and *Myogenin*, with concurrent attenuation of inflammatory signaling, reinforcement of antioxidant defense, and attenuation of pro-apoptotic signaling in this DEX-induced model.

Taxifolin-3-O-arabinopyranoside (Tax-G), identified as a major marker flavonoid of RMB, may contribute to these multi-target effects based on its known biological properties and our previous in vitro findings; however, its causal contribution in vivo remains to be established using the isolated compound. By systematically evaluating key muscle-related biomarkers, including Akt, mTOR, FoxO3α, Bax, Bcl-2, *Myostatin*, *Atrogin-1*, and *MuRF1*, the present study provides preclinical evidence relevant to the regulatory-science development of RMB as a functional ingredient. Together with our previous in vitro findings [43], these results support further investigation of RMB as a natural functional material for the prevention or management of muscle atrophy and age-related muscle loss, with validation in aged-animal models and, ultimately, clinical studies warranted.

## 6. Patents

The corresponding author (S.E.C.) declares filing patents related to the use of Taxifolin from *Rhododendron mucronulatum* in compositions for treating or alleviating muscle loss and muscle atrophy. Prof. Dr. Sun Eun Choi has patents #KR10-2022-0004024, #KR10-2811186, #PCT/KR2022/006679, #2024-541228, #202280088465.X, #1-2024-05102, #18/728,029 and #19/252,524 licensed to Prof. Dr. Sun Eun Choi.

## Figures and Tables

**Table 1:** Test group composition.

	G1	G2	G3	G4	G5
Dexamethasone Administration	-	+	+	+	+
RMB (mg/kg BW)	-	-	100	200	400
Number of Experimental Animals	10	10	10	10	10

RMB: *R. mucronulatum* branch extract (Lot No. DJTH-05572). -, not administered; +, administered.

**Table 2:** Information regarding the antibodies used in the experiments.

Antibody	Details	Manufacturer	Dilution
Antibody: phospho-mTOR (Ser2448)	#5536	Cell Signaling Technology	1:1000
Antibody: mTOR	#2972	Cell Signaling Technology	1:1000
Antibody: phospho-FoxO3α (Ser253)	#9466	Cell Signaling Technology	1:1000
Antibody: FoxO3α	#2497	Cell Signaling Technology	1:1000
Antibody: phospho-Akt (Ser473)	#4051	Cell Signaling Technology	1:1000
Antibody: Akt	#9272	Cell Signaling Technology	1:1000
Antibody: Bax	#2772	Cell Signaling Technology	1:1000
Antibody: Bcl-2	#3498	Cell Signaling Technology	1:1000
Antibody: Actin	#3700	Cell Signaling Technology	1:1000
Antibody: HRP-linked anti-rabbit IgG	#7074S	Cell Signaling Technology	1:5000
Antibody: HRP-linked anti-mouse IgG	#7076S	Cell Signaling Technology	1:5000

Primary antibodies were diluted in 5% BSA in TBST, and secondary antibodies were diluted in 5% nonfat dry milk in TBST. #, catalog number.

**Table 3:** Primer sequences for real-time PCR used in this study.

mRNA	Primer Sequences		GenBank No.
	Forward	Reverse	
*Atrogin* *-* *1*	5′-GCCCTCCACACTAGTTGACC-3′	5′-GACGGATTGACAGCCAGGAA-3′	NM_026346.3
*Catalase*	5′-GAACGAGGAGGAGAGGAAAC-3′	5′-TGAAATTCTTGACCGCTTTC-3′	NM_009804.2
*GPx1*	5′-CGCTCTTTACCTTCCTGCGGAA-3′	5′-AGTTCCAGGCAATGTCGTTGCG-3′	NM_001329528.1
*IGF-1*	5′-GTGGATGCTCTTCAGTTCGTGTG-3′	5′-TCCAGTCTCCTCAGATCACAGC-3′	NM_184052
*IL-1β*	5′-TGGACCTTCCAGGATGAGGACA-3′	5′-GTTCATCTCGGAGCCTGTAGTG-3′	NM_008361
*IL-6*	5′-CCTCTGGTCTTCTGGAGTACC-3′	5′-ACTCCTTCTGTGACTCCAGC-3′	NM_031168.2
*M* *u* *RF1*	5′-GAGGGCCATTGACTTTGGGA-3′	5′-TTTACCCTCTGTGGTCACGC-3′	NM_001039048.2
*MyoD1*	5′-GCACTACAGTGGCGACTCAGAT-3′	5′-TAGTAGGCGGTGTCGTAGCCAT-3′	NM_010866.2
*Myogenin*	5′-CCATCCAGTACATTGAGCGCCT-3′	5′-CTGTGGGAGTTGCATTCACTGG-3′	NM_031189.2
*Myostatin*	5′-ACTGGACCTCTCGATAGAACACTC-3′	5′-ACTTAGTGCTGTGTGTGTGGAGAT-3′	NM_010834.2
*SOD2*	5′-ATCAGGACCCATTGCAAGGA-3′	5′-AGGTTTCACTTCTTGCAAGCT-3′	NM_013671.3
*TNF-α*	5′-GGTGCCTATGTCTCAGCCTCTT-3′	5′-GCCATAGAACTGATGAGAGGGAG-3′	NM_013693
*GAPDH*	5′-TGGGTGTGAACCATGAGAAG-3′	5′-GCTAAGCAGTTGGTGGTGC-3′	NM_008084.3

## Data Availability

The original contributions presented in this study are included in the article/Supplementary Materials. Further inquiries can be directed to the corresponding author.

## Supplementary Materials

The following supporting information can be downloaded at: https://www.mdpi.com/article/10.3390/ijms27219811/s1.

## Abbreviations

Akt	Protein kinase B
ANOVA	Analysis of variance
Atrogin-1	Muscle atrophy F-box
Bax	Bcl-2-associated X protein
BCA	Bicinchoninic acid
Bcl-2	B-cell lymphoma 2
BSA	Bovine serum albumin
BW	Body weight
cDNA	Complementary DNA
CSA	Cross-sectional area
DEX	Dexamethasone
DEXA	Dual-energy X-ray absorptiometry
DTNB	5,5′-Dithiobis(2-nitrobenzoic acid)
EDL	Extensor digitorum longus
FoxO3α	Forkhead box O3α
GA	Gastrocnemius
GAPDH	Glyceraldehyde-3-phosphate dehydrogenase
GHS	Globally Harmonized System of Classification and Labelling of Chemicals
GLP	Good Laboratory Practice
GMP	Good Manufacturing Practice
GPx	Glutathione peroxidase
GPx1	Glutathione peroxidase 1
GSH	Reduced glutathione
GSSG	Oxidized glutathione
H&E	Hematoxylin and eosin
HPLC	High-performance liquid chromatography
HRP	Horseradish peroxidase
IACUC	Institutional Animal Care and Use Committee
IGF-1	Insulin-like growth factor-1
IL-1β	Interleukin-1β
IL-6	Interleukin-6
MFDS	Ministry of Food and Drug Safety
mTOR	Mammalian target of rapamycin
MuRF1	Muscle RING finger-1
MyoD	Myogenic differentiation 1
NF-κB	Nuclear factor-κB
NOAEL	No-observed-adverse-effect level
p70S6K	p70 ribosomal S6 kinase
PARP	Poly(ADP-ribose) polymerase
PBS	Phosphate-buffered saline
PCR	Polymerase chain reaction
PFA	Paraformaldehyde
PI3K	Phosphoinositide 3-kinase
QF	Quadriceps femoris
RMB	*Rhododendron mucronulatum* branch extract
ROS	Reactive oxygen species
SDS-PAGE	Sodium dodecyl sulfate polyacrylamide gel electrophoresis
SEM	Standard error of the mean
SOD	Superoxide dismutase
SOD2	Superoxide dismutase 2
SOL	Soleus
SPF	Specific pathogen-free
TA	Tibialis anterior
Tax-A	Taxifolin aglycone
Tax-G	Taxifolin-3-O-arabinopyranoside
TBST	Tris-buffered saline containing Tween 20
TGF-β	Transforming growth factor-β
TLC	Thin-layer chromatography
TNB	5-Thio-2-nitrobenzoic acid
TNF-α	Tumor necrosis factor-α
