# Supplementary material for: Effects of Rhododendron mucronulatum Branch Extract on Dexamethasone-Induced Muscle Loss and Muscle Atrophy in C57BL/6 Mice
Source: Int J Mol Sci. 2026 Nov 1;27(21):9811. doi: 10.3390/ijms27219811 (PMC13763443; doi:10.3390/ijms27219811)
Supplement: Supplementary file 1 [file ijms-27-09811-s001.zip › ijms-4582420-supplementary.pdf]

Table S1. Results of the Shapiro–Wilk normality test, one-way ANOVA, and Kruskal–Wallis test for all parameters.

Parameter	*n* (G1/G2/G3/G4/G5)	Shapiro–Wilk test, *p*					One-way ANOVA		Kruskal–Wallis test	
		G1	G2	G3	G4	G5	*F* (df)	*p*	*H*	*p*
Body weight (Figure 1)										
Body weight, week 0 (g)	10/10/10/10/10	0.526	0.866	0.446	0.210	0.069	0.12 (4, 45)	0.976	–	–
Body weight, week 1 (g)	10/10/10/10/10	0.568	0.734	0.417	0.912	0.507	0.67 (4, 45)	0.613	–	–
Body weight, week 2 (g)	10/10/10/10/10	0.621	0.935	0.383	0.557	0.654	1.49 (4, 45)	0.222	–	–
Body weight, week 3 (g)	10/10/10/10/10	0.829	0.388	0.116	0.529	0.982	4.93 (4, 45)	0.002	–	–
Body weight, week 4 (g)	10/10/10/10/10	0.245	0.386	0.560	0.588	0.310	6.95 (4, 45)	< 0.001	–	–
Physical performance and body composition (Figure 2)										
Time to exhaustion (min)	10/10/10/10/10	0.858	**0.037**	**0.040**	0.849	0.098	32.33 (4, 45)	< 0.001	38.743	< 0.001
Exercise capacity (J)	10/10/10/10/10	0.606	0.055	0.077	0.729	0.116	33.21 (4, 45)	< 0.001	–	–
Grip strength (g)	10/10/10/10/10	0.710	0.180	0.527	0.377	**0.010**	15.40 (4, 45)	< 0.001	26.678	< 0.001
Body fat (%)	10/10/10/10/10	0.991	0.084	**0.037**	0.847	0.996	5.34 (4, 45)	0.001	15.654	0.004
Lean body (%)	10/10/10/10/10	0.991	0.084	**0.037**	0.847	0.996	5.34 (4, 45)	0.001	15.654	0.004
Muscle weight (Figure 3)										
QF weight (g)	10/10/10/10/10	0.383	0.468	0.582	0.315	0.385	2.14 (4, 45)	0.091	–	–
GA weight (g)	10/10/10/10/10	0.292	0.123	0.909	0.682	0.765	9.22 (4, 45)	< 0.001	–	–
SOL weight (g)	10/10/10/10/10	0.741	0.799	0.733	0.516	0.246	2.33 (4, 45)	0.070	–	–
EDL weight (g)	10/10/10/10/10	0.264	0.336	0.169	0.083	0.247	4.92 (4, 45)	0.002	–	–
TA weight (g)	10/10/10/10/10	**0.039**	0.755	0.222	0.062	0.496	2.27 (4, 45)	0.076	5.954	0.203
Relative QF weight (g/100 g BW)	10/10/10/10/10	0.100	0.474	0.598	0.241	0.628	1.33 (4, 45)	0.275	–	–
Relative GA weight (g/100 g BW)	10/10/10/10/10	0.063	0.615	0.314	0.450	0.585	3.01 (4, 45)	0.028	–	–
Relative SOL weight (g/100 g BW)	10/10/10/10/10	0.271	0.969	0.660	0.311	0.636	0.26 (4, 45)	0.902	–	–
Relative EDL weight (g/100 g BW)	10/10/10/10/10	0.341	0.423	0.362	0.277	0.637	2.57 (4, 45)	0.051	–	–
Relative TA weight (g/100 g BW)	10/10/10/10/10	0.151	0.368	0.321	0.497	0.544	0.96 (4, 45)	0.438	–	–
Muscle fiber cross-sectional area (Figure 4)										
CSA (a.u.)	10/10/10/10/10	0.404	0.937	0.824	0.886	0.155	15.71 (4, 45)	< 0.001	–	–
Protein levels in GA (Figure 5)										
Phospho-Akt	12/10/10/10/10	0.452	0.173	0.937	0.084	0.090	3.24 (4, 47)	0.020	–	–
Akt	12/10/10/10/10	0.292	0.903	0.182	0.068	0.533	4.83 (4, 47)	0.002	–	–
p-Akt/Akt ratio	12/10/10/10/10	0.602	0.988	**0.034**	0.780	0.770	2.80 (4, 47)	0.036	12.781	0.012
Phospho-mTOR	12/10/10/10/10	0.231	0.067	0.772	0.912	0.065	5.57 (4, 47)	< 0.001	–	–
mTOR	12/10/10/10/10	0.659	0.710	0.480	0.382	**0.021**	3.09 (4, 47)	0.025	11.772	0.019
p-mTOR/mTOR ratio	12/10/10/10/10	0.394	0.139	**0.046**	0.657	0.425	6.46 (4, 47)	< 0.001	20.537	< 0.001
Phospho-FoxO3α	12/10/10/10/10	0.257	0.611	0.570	0.724	0.587	5.48 (4, 47)	0.001	–	–
FoxO3α	12/10/10/10/10	0.240	0.057	0.847	0.303	0.604	37.31 (4, 47)	< 0.001	–	–
p-FoxO3α/FoxO3α ratio	12/10/10/10/10	0.131	0.398	**0.026**	**0.026**	**0.042**	4.66 (4, 47)	0.003	22.866	< 0.001
Bcl-2	12/10/10/10/10	0.971	0.337	**0.012**	0.258	0.130	16.61 (4, 47)	< 0.001	28.338	< 0.001
Bax	12/10/10/10/10	**0.014**	0.245	0.743	**0.012**	0.794	2.23 (4, 47)	0.080	8.476	0.076

Parameter	n (G1/G2/G3/G4/G5)	Shapiro–Wilk test, p					One-way ANOVA		Kruskal–Wallis test	
		G1	G2	G3	G4	G5	F (df)	p	H	p
mRNA expression in SOL (Figure 6)										
MyoD	6/6/6/6/6	0.242	0.612	0.277	**0.020**	0.872	9.35 (4, 25)	< 0.001	19.879	< 0.001
Myogenin	6/6/6/6/6	0.528	0.139	**0.018**	0.704	0.086	3.03 (4, 25)	0.036	11.160	0.025
IGF-1	6/6/6/6/6	0.971	0.462	0.273	0.319	0.849	16.33 (4, 25)	< 0.001	–	–
Myostatin	6/6/6/6/6	0.580	0.513	**0.013**	0.557	0.305	3.95 (4, 25)	0.013	14.841	0.005
Atrogin-1	6/6/6/6/6	0.205	0.305	0.274	0.120	0.643	11.39 (4, 25)	< 0.001	–	–
MuRF1	6/6/6/6/6	0.630	0.309	0.149	**0.034**	0.300	5.01 (4, 25)	0.004	14.759	0.005
IL-1β	6/6/6/6/6	0.104	0.113	0.522	0.310	0.577	4.83 (4, 25)	0.005	–	–
IL-6	6/6/6/6/6	0.729	0.681	0.533	0.437	**0.028**	7.73 (4, 25)	< 0.001	18.764	< 0.001
TNF-α	6/6/6/6/6	**0.010**	0.277	**0.016**	0.920	0.072	12.27 (4, 25)	< 0.001	20.546	< 0.001
SOD2	6/6/6/6/6	0.135	0.110	0.433	0.076	0.798	9.97 (4, 25)	< 0.001	–	–
Catalase	6/6/6/6/6	0.212	0.766	0.414	0.700	0.488	4.36 (4, 25)	0.008	–	–
GPx1	6/6/6/6/6	0.953	0.537	0.362	0.657	0.145	4.88 (4, 25)	0.005	–	–
Serum antioxidant status (Figure 7)										
Glutathione (μM)	10/10/10/10/10	0.739	0.728	0.471	0.155	0.967	8.70 (4, 45)	< 0.001	–	–
SOD activity (U/mL)	10/10/10/10/10	0.426	0.210	0.751	0.703	0.859	11.68 (4, 45)	< 0.001	–	–
Catalase activity (nmol/min/mL)	10/10/10/10/10	0.231	**0.005**	0.931	**0.013**	0.188	3.60 (4, 45)	0.012	10.130	0.038
GPx activity (nmol/min/mL)	10/10/10/10/10	**0.019**	0.397	0.468	0.728	0.913	5.98 (4, 45)	< 0.001	16.920	0.002

Shapiro–Wilk p-values < 0.05 (deviation from normality) are shown in bold. Of the 240 group-wise tests, 215 (89.6%) showed no significant deviation from normality.

The Kruskal–Wallis test was performed for the 19 parameters in which at least one group deviated from normality; –, not performed. For all 19 parameters, the Kruskal–Wallis test yielded the same conclusion (significant or not significant at $p < 0.05$) as the one-way ANOVA.

When the one-way ANOVA was significant, predefined pairwise comparisons (G2 vs. G1 and G3–G5 vs. G2) were performed with Bonferroni correction for four comparisons; the results are shown in Figures 1–7.

G1, normal control; G2, DEX control; G3–G5, DEX + RMB 100, 200, and 400 mg/kg; df, degrees of freedom (between groups, within groups); QF, quadriceps femoris; GA, gastrocnemius; SOL, soleus; EDL, extensor digitorum longus; TA, tibialis anterior; BW, body weight; CSA, cross-sectional area.
